# Comparative Analyses of Van Nuys Prognostic Index and NCCN Guidelines in Ductal Carcinoma *In Situ* Treatment in a Brazilian Hospital

**DOI:** 10.3390/life15030432

**Published:** 2025-03-09

**Authors:** Marcelo Antonini, Raissa Barros Vasconcelos, André Mattar, Mariana Pollone Medeiros, Marina Diógenes Teixeira, Andressa Gonçalves Amorim, Odair Ferraro, Larissa Chrispim de Oliveira, Marcellus do Nascimento Moreira Ramos, Francisco Pimentel Cavalcante, Felipe Zerwes, Marcelo Madeira, Eduardo de Camargo Millen, Antonio Luiz Frasson, Fabricio Palermo Brenelli, Gil Facina, Henrique Lima Couto, Luiz Henrique Gebrim

**Affiliations:** 1 Department of Breast Surgery, Hospital do Servidor Público Estadual Francisco Morato de Oliveira, Sao Paulo 04039-000, SP, Brazil; draraissa.mastologia@gmail.com (R.B.V.); marianapollone@gmail.com (M.P.M.); odairferraro@hotmail.com (O.F.); 2 Centro de Desenvolvimento de Ensino e Pesquisa do Instituo de Assistência Médica ao Servidor Público Estadual (CEDEP-IAMSPE), Sao Paulo 04039-000, SP, Brazil; 3 BBREAST—Brazilian Breast Association Team, Sao Paulo 01258-011, SP, Brazil; mattar.andre@gmail.com; 4 Department of Breast Surgery, Centro de Referência da Saúde da Mulher—Hospital da Mulher, Sao Paulo 01206-001, SP, Brazil; mari_diogenes@hotmail.com (M.D.T.); andressaamorim88@hotmail.com (A.G.A.); chrispiml@hotmail.com (L.C.d.O.); marcellusnmr@hotmail.com (M.d.N.M.R.); 5 Department of Breast Surgery, Hospital Geral de Fortaleza, Fortaleza 60150-160, CE, Brazil; fpimentelcavalcante@gmail.com; 6 Department of Breast Surgery, Medical School of the Pontifícia Universidade Católica do Rio Grande do Sul (PUCRS), Porto Alegre 90619-900, RS, Brazil; zerwes@hotmail.com; 7 Department of Breast Surgery, Oncoclínicas, Porto Alegre 90570-020, RS, Brazil; 8 Department of Breast Surgery, Faculdade Israelita de Ciências da Saúde Albert Einstein, Sao Paulo 05653-000, SP, Brazil; marcemadeira@gmail.com; 9 Department of Breast Surgery, Americas Oncologia, Rio de Janeiro 22793-080, RJ, Brazil; eduardomillen@gmail.com; 10 Department of Breast Surgery, Hospital Albert Einstein, Sao Paulo 05651-901, SP, Brazil; alfrasson.af@gmail.com; 11 Department of Breast Surgery, Universidade Estadual de Campinas (UNICAMP), Campinas 13082-859, SP, Brazil; fabriciobrenelli@hotmail.com; 12 Department of Gynecology, Universidade Federal de Sao Paulo, Sao Paulo 04023-062, SP, Brazil; facina@unifesp.br; 13 Department of Breast Surgery, REDIMAMA-REDIMASTO, Belo Horizonte 30110-022, MG, Brazil; enriquecouto@hotmail.com; 14 Department of Breast Surgery, Hospital Beneficiencia Portuguesa, Sao Paulo 01438-000, SP, Brazil; lgebrim1964@gmail.com

**Keywords:** ductal carcinoma *in situ*, invasive breast cancer, DCIS treatment, Van Nuys Prognostic Index, National Comprehensive Cancer Network

## Abstract

Background: Ductal carcinoma *in situ* (DCIS) is a precursor of invasive breast cancer and its early diagnosis and treatment are essential to prevent progression and recurrences. Risk stratification guidelines, such as the Van Nuys Prognostic Index (VNPI) and those by the National Comprehensive Cancer Network (NCCN), help guide appropriate treatment. This study compares VNPI recommendations for DCIS patients treated at Hospital do Servidor Público Estadual de São Paulo (HSPE) with NCCN guidelines, focusing on treatment conducted and recurrence rates. Methods: This retrospective, cross-sectional study reviewed medical records of 145 patients treated for DCIS at HSPE between January 1996 and June 2022, with a mean follow-up of 60.3 months. Results: Based on VNPI, 38.8% were low risk, 53.2% intermediate risk, and 7.8% high risk. NCCN guidelines classified only 12.9% as low risk and 87.1% as high risk. Treatment included breast-conserving surgery (BCS) with radiotherapy (43.1%), BCS alone (38.8%), and mastectomy (18.1%). There were 18 recurrences (15.5%): 5.2% as DCIS and 10.3% as invasive cancer. Of these recurrences, 5.6% occurred in patients who, according to NCCN, would have received BCS with radiotherapy or mastectomy. Conclusion: By integrating the VNPI with NCCN treatment guidelines, the NCCN’s recommendations could potentially reduce local recurrence rates by 5.6%. However, further studies are necessary to evaluate the long-term impact of these guidelines on overall survival outcomes.

## 1. Introduction

Breast cancer is the most common malignant neoplasia among women in Brazil and worldwide, excluding non-melanoma skin cancer. An estimated 73,610 new cases are expected in Brazil in 2025, including cases of invasive carcinoma (IBC) and ductal carcinoma *in situ* (DCIS) [1]. DCIS is considered a precursor lesion for IBC, currently accounting for over 20% of new breast cancer diagnoses in the United States, with more than 60,000 cases diagnosed annually [2].

DCIS is a breast neoplasia originating from a terminal duct-lobular unit, characterized by the proliferation of ductal epithelial cells that grow in different architectural patterns [3,4]. This growth is confined to the duct-lobular unit, without extending beyond the myoepithelial layer’s basement membrane, and therefore does not lead to stromal invasion [2]. The incidence of DCIS has been rising, primarily due to increased access to mammographic screening [2,5].

DCIS is a heterogeneous entity in terms of presentation, morphology, biomarker expression, genetic alterations, and natural history [6,7]. Most cases are asymptomatic or present with small palpable nodules. Radiologically, 75% of lesions appear as calcifications, and some may go undetected by mammography. Additionally, the area of microcalcifications on imaging may underestimate the true lesion size, which can result in incomplete excision in breast-conserving surgeries [2,8].

Recommended treatment options include mastectomy with sentinel lymph node biopsy, breast-conserving surgery alone or followed by radiotherapy, with the potential addition of hormonal therapy with Tamoxifen or Aromatase Inhibitors in postmenopausal patients for DCIS cases that express hormone receptors [2,9].

Although DCIS is not an invasive disease, its potential for progression underscores the critical importance of timely diagnosis and appropriate treatment [7]. However, many of these lesions have an indolent behavior and may not progress to invasive breast carcinoma (IBC) or may take years to do so [2]. Small retrospective studies have shown that 30% of DCIS cases may progress to IBC over a period of up to 30 years [7]. Thus, predicting the progression of these lesions to invasive disease remains a challenge, as does planning proportionate treatment to avoid overtreatment [5].

Despite its favorable prognosis and lack of impact on life expectancy, a DCIS diagnosis can result in psychological stress [5,6] and its treatment may lead to procedure-related morbidities, work and social activity limitations, as well as changes in self-image [5,10]. To address these concerns, risk calculators and guidelines have been developed as tools for risk estimation and to aid in decision-making following a DCIS diagnosis [11].

In 1996, Silverstein introduced the Van Nuys Prognostic Index (VNPI), which was later updated in 2003, to evaluate prognosis and guide the management of DCIS. The VNPI stratifies the risk of ipsilateral DCIS recurrence by assessing lesion size, margin distance in the surgical specimen, nuclear grade, presence of necrosis, and patient age [2]. Designed to support decision-making, it helps determine the most suitable approach—whether radical surgery, breast-conserving surgery alone, or combined with radiotherapy—while aiming to achieve a local recurrence rate below 20% over 12 years [12,13]. Each variable is assigned a score from 1 to 3. Patients scoring 4–6 are considered low risk, those scoring 7–9 as intermediate risk, and those scoring 10–12 as high risk [12,14].

According to the VNPI study, low-risk patients could undergo wide excision without adjuvant radiotherapy. Intermediate-risk patients would benefit from the inclusion of adjuvant radiotherapy to reduce recurrence rates. Finally, high-risk patients would be candidates for mastectomy, as they exhibited higher recurrence rates even with radiotherapy [2,14].

The National Comprehensive Cancer Network (NCCN) is a nonprofit alliance of U.S. cancer centers that periodically releases guidelines for the diagnosis, treatment, and follow-up of various cancers. NCCN-recommended treatment options for DCIS include mastectomy with sentinel lymph node biopsy, conservative surgery with adjuvant radiotherapy, or isolated conservative surgery with possible addition of Tamoxifen and Aromatase Inhibitors in postmenopausal women with hormone receptor-positive DCIS [2,9].

The NCCN on its 2024 version suggests that only selected patients with low-risk DCIS could be treated with surgery followed by partial breast radiation or even omission of adjuvant radiotherapy, provided they meet the following criteria: detection through screening, low or intermediate nuclear grade, tumor size less than 2.5 cm, and a surgical margin clearance of at least 3 mm [9].

The application of the VNPI and other guidelines varies across different countries and institutions, underscoring the importance of assessing each calculator’s effectiveness [11]. Several studies have evaluated the predictive value of VNPI and NCCN criteria in DCIS treatment [11,12,13]. However, most focus on isolated guideline recommendations rather than a direct comparison between them. Additionally, there are limited data from non-Western populations, such as the Brazilian cohort in our study. This study aims to address this gap by comparing recurrence rates and treatment strategies between VNPI and NCCN classifications.

## 2. Materials and Methods

### 2.1. Study Design

We conducted a retrospective observational cross-sectional study by reviewing medical records from the Breast Surgery Department of the Hospital do Servidor Público Estadual (HSPE), Brazil, for patients who underwent surgical treatment for breast cancer (BC) between January 1996 and June 2022.

### 2.2. Inclusion and Exclusion Criteria

Female patients over 18 years of age, diagnosed with ductal carcinoma in situ DCIS, and treated according to the Van Nuys Prognostic Index (VNPI) were included. We excluded all patients with invasive ductal carcinoma, patients with metastasis, and patients without follow up or incomplete data.

Incomplete data were excluded from the study if critical clinical and pathological variables were missing, including tumor size, nuclear grade, margin status, or recurrence data. However, patients with missing secondary data, such as hormone receptor status or HER-2 expression, were retained in the analysis and the missing values were reported in the results.

### 2.3. Data Source

The medical records used in this study were retrieved from an institutional database containing all patients who underwent surgical treatment, stored in an Excel spreadsheet. Data extraction was performed manually and cross-checked with individual patient records to ensure accuracy and completeness.

Limitations of retrospective studies, including the possibility of missing data and information bias. To mitigate these issues, we cross-checked data across multiple sources, including electronic and physical medical records, to ensure data accuracy and completeness.

The study included patients diagnosed and treated for ductal carcinoma in situ (DCIS) at the Hospital do Servidor Público Estadual de São Paulo (HSPE) between January 1996 and June 2022. Given the extended time frame of the study, we accounted for changes in clinical practice by stratifying data analysis based on treatment era, ensuring consistency in follow-up and medical record documentation.

### 2.4. Van Nuys Prognostic Index and Treatment

The VNPI is presented in the Table 1.

According to their classification under the VNPI, patients underwent one of three treatments: breast-conserving surgery (BCS) alone, breast-conserving surgery with adjuvant radiotherapy (RT), or mastectomy. Recurrence rates were calculated based on the treatment performed. Next, recurrences were assessed in the low-risk and high-risk groups of patients if classified according to NCCN recommendations.

### 2.5. Follow-Up Duration

Follow-up duration was standardized across patients, with clinical evaluations every 6 months for the first 5 years and annually thereafter. Imaging follow-up was performed per institutional guidelines. Any variations in post-treatment monitoring were accounted for in the statistical analysis to minimize bias.

### 2.6. Statistical Analysis

Data recording and statistical analysis were performed using Excel spreadsheets and SPSS 22.0 for Windows. Initially, all variables were analyzed descriptively. For quantitative variables, this analysis involved observing minimum values, and the comparison of means between two groups was carried out using Student’s *t*-test. When the normality assumption was rejected, the non-parametric Mann–Whitney test was used.

To test homogeneity between proportions, the Chi-square test or Fisher’s exact test (when expected frequencies were less than 5) was applied. Calculations were conducted using SPSS 22.0 for Windows, with a 5% significance level for the tests.

Multivariate logistic regression analysis was performed to identify independent predictors of recurrence, adjusting for age, nuclear grade, tumor size, margin status, and treatment type. Bonferroni correction was not applied, as multiple hypothesis testing was limited to predefined primary endpoints.

### 2.7. Ethics

The study was submitted to and approved by the Research Ethics Committee of the Hospital do Servidor Público Estadual-Francisco Morato de Oliveira and registered on the Brazil Platform with approval number CAAE: 60175722.6.0000.5463. The need for informed consent was waived due to the retrospective nature of the study and the confidentiality and integrity of the medical record data were preserved.

## 3. Results

**Figure 1:**
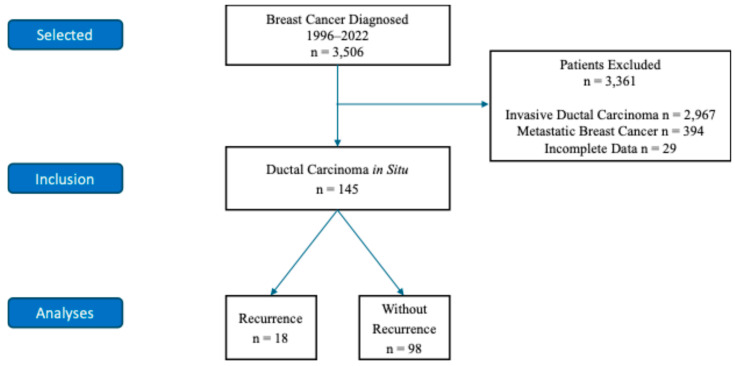
Flowchart of included patients with DCIS.

From January 1996 to June 2022, 3.216 patients were diagnosed with breast cancer at HSPE, of which 394 (12.5%) had metastatic disease, 2.967 (83.9%) had stage I to III breast cancer, and 145 (3.6%) had ductal carcinoma in situ (DCIS). Among the DCIS patients, 29 patients were excluded due to missing critical data. Specifically, 12 cases lacked tumor size information, 8 cases had missing margin status, 5 cases had missing nuclear grade, and 4 cases had no recurrence data available. We can see those data at Figure 1.

### 3.1. Epidemiological Profile

The mean age of the 116 included patients was 60.1 years, with a standard deviation of 11.0 years. Among them, 26 (22.4%) were premenopausal and 90 (77.6%) were postmenopausal (*p* < 0.001). Regarding family history of breast cancer in first-degree relatives, only 16 (13.8%) had a family history, while 100 (86.2%) did not (*p* < 0.001).

The diagnosis was made through mammographic screening in 88 patients (75.9%) and clinically in 28 patients (24.1%) (*p* < 0.001). The histological type was ductal carcinoma in situ, with comedonecrosis present in 70 patients (60.3%) and high-grade lesions in 45 patients (38.8%) (*p* < 0.05).

The size of the lesions found was up to 1.5 cm in 64 patients (39.7%), and margins were larger than 10 mm in 44 patients (41.5%), with lesion size showing a significant difference (*p* = 0.006) and margins showing no significant difference (*p* = 0.269).

Immunohistochemistry was unknown in 64 patients (53.2%). Among those for whom it was known, 44 patients (37.9%) had positive hormone receptors, and 8 (6.9%) had negative hormone receptors, with a significant difference (*p* < 0.001). For HER-2 positivity, eight patients (6.9%) were positive, showing a significant difference (*p* = 0.008).

The type of treatment was breast-conserving surgery in 45 patients (38.8%), breast-conserving surgery with adjuvant radiotherapy in 50 (43.1%), and mastectomy in 21 patients (18.1%), with a significant difference (*p* < 0.001). Due to the extended follow-up period, there was a time when sentinel lymph node biopsy (SLNB) was not performed on DCIS patients undergoing mastectomy. Thus, SLNB was performed in 15 patients: 10 (47.6%) who underwent mastectomy and 5 (5.2%) who underwent breast-conserving surgery, with a significant difference (*p* < 0.001).

The mean follow-up time for the patients was 60.3 months, with a standard deviation of 47.4 months, a maximum of 203 months, and a minimum of 6 months.

Table 2 shows the distributions of the quantitative variables and Table 3 displays the qualitative variables of the epidemiological profile.

### 3.2. Recurrence

During the follow-up, 18 (15.5%) recurrences were observed, with 6 (5.2%) due to DCIS and 12 (10.3%) due to IBC, showing a significant difference (*p* < 0.001). The mean time to recurrence was 20.9 months, with a standard deviation of 17.6 months, and a maximum time of 73 months and a minimum of 2 months.

A log-rank test was performed to compare recurrence-free survival between invasive and in situ recurrences. The results showed no statistically significant difference between the two survival curves (χ^2^ = 0.0, *p* = 1.0). This indicates that the recurrence rates over time for both invasive and in situ groups were similar in this cohort.

**Figure 2:**
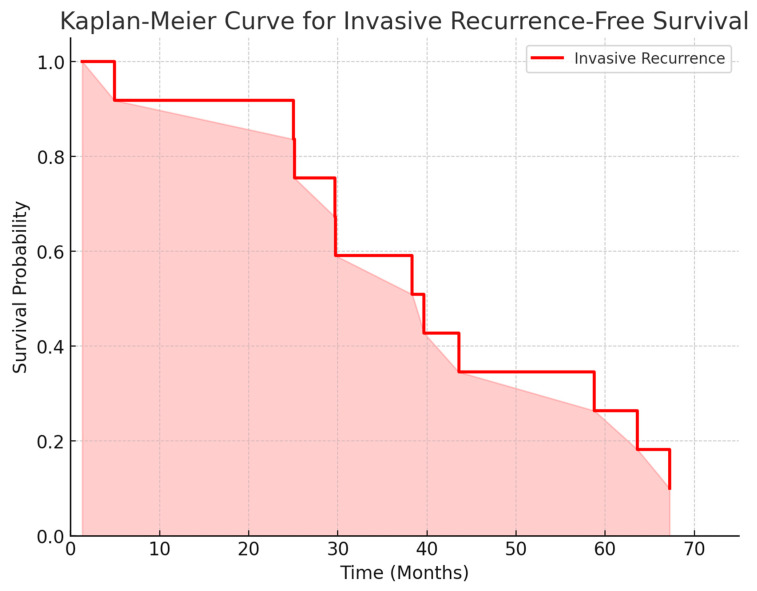
Kaplan–Meier curve for invasive recurrence-free survival.

**Figure 3:**
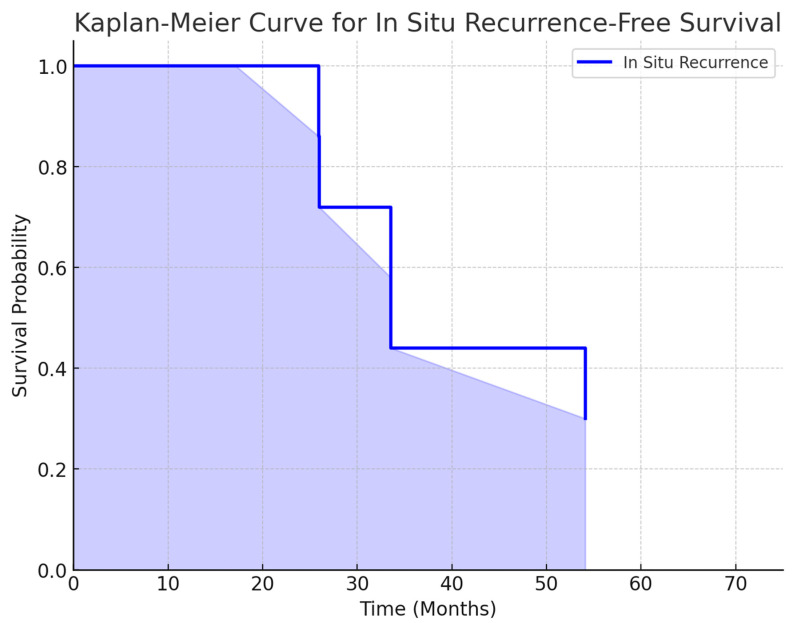
Kaplan–Meier curve for *in situ* recurrence-free survival.

A Cox proportional hazards model was applied to compare the recurrence-free survival between invasive and in situ recurrences. The results showed that in situ recurrences had a higher risk over time (HR = 1.87; 95% CI: 0.69–5.12; *p* = 0.220). However, this difference was not statistically significant, indicating that both types of recurrence may follow a similar time-to-event pattern in this cohort. Figure 2 and Figure 3 we demonstrate the in situ and invasive recurrence curves.

### 3.3. Evaluation of Recurrences According to IPVN and NCCN Guidelines

The patients classified as low risk according to the IPVN were 45 (38.8%), intermediate risk 62 (53.2%), and high risk 9 (7.8%). The patients classified as low risk according to the NCCN were 15 (12.9%) and high risk 101 (87.1%). In Table 4 we find the distribution according to VNPI classification, treatment, and NCCN classification.

The recurrences according to the treatment approach guided by the IPVN were 10 (55.6%) among patients who underwent isolated conservative surgery, 7 (38.9%) among those who underwent surgery followed by radiotherapy, and 1 (5.6%) among those who underwent mastectomy.

The VNPI classified 7.8% of patients as high risk, whereas the NCCN guidelines classified 87.1% in this category. The recurrence rate among patients classified as low or intermediate risk by VNPI was 55.6%, whereas it was 16.7% for patients classified as high risk under NCCN criteria. These findings suggest that VNPI underestimates recurrence risk in some patients, leading to potential undertreatment.

Among recurrences, 17 (94, 4%) patients were treated in accordance with NCCN classification and 1 (5, 6%) was not treated according to NCCN recommendations. In Table 5 we find the recurrence according to NCCN- and VNPI-based treatments.

Among patients classified as NCCN high risk, 94.4% (17/18) experienced recurrence, compared to 85.7% (84/98) in the NCCN low-risk group (RR = 2.09, 95% CI: 0.81–5.37, *p* = 0.156). The NCCN low-risk subgroup exhibited a recurrence rate of 5.6% (1/18) versus 14.3% (14/98) in the non-recurrent group, with an RR of 0.83 (95% CI: 0.10–6.56, *p* = 0.999).

Regarding VNPI subgroups, the low VNPI group had a recurrence rate of 55.6% (10/18), with a relative risk of 2.76 (95% CI: 1.01–7.51, *p* = 0.049), compared to the intermediate VNPI subgroup, which showed a recurrence rate of 27.7% (5/18) and an RR of 0.36 (95% CI: 0.13–0.99, *p* = 0.049). The high VNPI group demonstrated the highest recurrence rate (16.7%, 3/18) with an RR of 4.13 (95% CI: 1.19–14.41, *p* = 0.058). In Table 6 we find the correlation of recurrence and VNPI and NCC subgroups.

These findings suggest that higher VNPI and NCCN risk groups are associated with an increased recurrence risk, whereas the intermediate VNPI subgroup presents a lower risk of recurrence. However, statistical significance was observed only for the VNPI low (*p* = 0.049) and intermediate subgroups (*p* = 0.049), indicating potential clinical relevance. 

### 3.4. Multivariate Logistic Regression

A multivariate logistic regression analysis was conducted to identify independent predictors of recurrence in DCIS patients. The results showed that age was significantly associated with an increased risk of recurrence (OR = 1.06; 95% CI: 1.00–1.12; *p* = 0.044), indicating a progressive rise in risk with advancing age. Margin status also showed a trend toward association with recurrence (OR = 1.55; 95% CI: 0.50–4.77), although it did not reach statistical significance (*p = 0.443*).

Nuclear grade (OR = 1.16; 95% CI: 0.55–2.44; *p* = 0.701) and tumor size (OR = 0.78; 95% CI: 0.44–1.39; *p* = 0.401) were not identified as independent risk factors. Treatment type, comparing breast-conserving surgery with mastectomy, showed a trend toward a lower risk with mastectomy (OR = 0.62; 95% CI: 0.18–2.13), but this result was not statistically significant (*p* = 0.447).

These findings on Table 7 suggest that age may be a relevant prognostic factor for recurrence, while the other analyzed variables did not demonstrate a statistically significant association when adjusted for competing factors.

## 4. Discussions

Several studies have aimed to characterize DCIS and IBC at the molecular level, revealing genetic similarities and likely common origins between the two conditions [2]. Research shows that approximately 30% of untreated DCIS cases will progress to IBC (about 30% to low- and intermediate-grade invasive lesions and around 60% to high-grade lesions within 5–20 years) [15]. However, estimating the progression risk of DCIS remains challenging due to the current standard of care, which generally involves excision with clear margins, radiotherapy, and/or adjuvant endocrine therapy when appropriate [2].

Randomized studies have demonstrated that adding radiotherapy to conservative surgery reduces local recurrence of DCIS, although the impact on overall survival or breast cancer-specific survival remains unclear. The primary role of radiotherapy in this context is to reduce the risk of invasive recurrences, which are associated with increased mortality [2]. While radiotherapy after conservative surgery has been shown to significantly reduce ipsilateral recurrence rates, adjuvant hormonal therapy reduces both ipsilateral and contralateral recurrence rates [16].

DCIS treatment requires a careful balance between preventing progression to invasive disease, avoiding recurrence, and minimizing potential overtreatment effects such as acute or chronic pain, fatigue, sensory disturbances, and other side effects, especially in patients with low-risk lesions [5].

In our study, 38.8% of patients were treated with isolated conservative surgery, and 43.1% underwent surgery combined with radiotherapy. Mastectomy was performed in 18.1% of the sample.

Several phase III trials are ongoing to assess the risk and benefit of active surveillance for low-risk DCIS, with main endpoints including ipsilateral invasive disease-free survival (LORIS), ipsilateral invasive breast cancer-free rate at 2 years (COMET), 5 years (LORETTA), and 10 years (LORD). Due to the lack of consensus on defining low-risk DCIS, these trials use various criteria, including age, tumor grade, lesion size, and other pathological features [15]. On the other hand, it is essential to consider that adopting active surveillance as a DCIS management alternative may lead to anxiety, psychological, and functional disorders in patients, as there is an awareness and fear of disease progression when left untreated [5,17,18,19,20].

Multiple factors may contribute to increased recurrence risk in DCIS, with complete lesion excision being the most critical [21]. Clear surgical margins (typically around 2 mm) are associated with reduced recurrence rates, as are low-grade tumors, absence of necrosis, lesion size less than 4 cm, and absence of clinical signs (such as palpable lesions or nipple discharge) [16,21]. These and other factors are used by some scores and guidelines to stratify the post-treatment recurrence risk.

The primary aim of this stratification is to identify low-risk patients who could benefit from less aggressive treatments.

The main guidelines and prognostic indices for DCIS include the Memorial Sloan Kettering Cancer Center Nomogram (MSKCC), University of Southern California Van Nuys Prognostic Index (VNPI), Oncotype DX DCIS, and the National Comprehensive Cancer Network (NCCN) prognostic index, each with limitations and none yet recognized as the optimal tool [11].

The VNPI determines DCIS recurrence risk and classifies patients based on age, tumor size, surgical margins, nuclear grade, and necrosis presence or absence. Low-risk patients are candidates for isolated conservative surgery, intermediate-risk patients for conservative surgery plus radiotherapy, and high-risk patients for mastectomy. The application of the VNPI varies significantly across countries and institutions; for example, over 70% of DCIS cases in Australia and New Zealand are managed according to the VNPI recommendations, compared to less than 16% in the United Kingdom [11]. However, the VNPI has limitations, including criteria changes over the years and the lack of external, independent validation [12,14,21].

VNPI places less emphasis on margin width and nuclear grade than NCCN, which may lead to under-classification of high-risk patients. For example, a patient with small margins (<2 mm) and high nuclear grade might still be categorized as intermediate risk under VNPI, while NCCN would classify them as high risk, leading to different treatment recommendations.

In turn, according to the 2024 NCCN version, whole-breast radiotherapy after conservative surgery reduces tumor recurrence rates by 50–70%. Only selected low-risk patients, particularly those eligible for adjuvant endocrine therapy, may be treated with isolated lesion excision, omitting radiotherapy. For low-risk classification, specific criteria must be met, such as routine screening detection (no clinical signs), low or intermediate nuclear grade, tumor size less than 2.5 cm, and margins greater than 3 mm [9].

A key difference between the VNPI and NCCN guidelines lies in their weighting of prognostic factors. The VNPI assigns numerical scores based on margin size, nuclear grade, tumor size, and patient age, categorizing patients into low, intermediate, or high-risk groups. NCCN guidelines, in contrast, rely more heavily on radiotherapy recommendations and classify nearly all cases as high risk unless strict low-risk criteria are met (e.g., margins >3 mm, tumor <2.5 cm). These differing approaches may contribute to under-classification of high-risk cases by VNPI, potentially leading to increased recurrence rates.

Our study demonstrates a significant difference in risk classification between VNPI and NCCN guidelines. While VNPI classified only 7.8% of patients as high risk, the NCCN guidelines categorized 87.1% in this group. This discrepancy is reflected in recurrence outcomes, where patients classified as low or intermediate risk by VNPI had a recurrence rate of 55.6%, while those classified as high risk under NCCN had a recurrence rate of 16.7%. This highlights the potential for VNPI to underestimate recurrence risk, possibly leading to suboptimal treatment recommendations compared to NCCN guidelines.

In our study sample, patients treated at HSPE were classified according to the VNPI, with 38.8% classified as low risk and thus managed with conservative surgery without adjuvant radiotherapy. In contrast, if classified according to NCCN criteria, only 12.9% would have been considered low risk and treated with surgery alone.

The impact of radiotherapy on DCIS treatment has been assessed in several studies. A systematic review and meta-analysis by Chen Q. et al. compared long-term outcomes of different treatment modalities for low-risk DCIS patients. It was concluded that even in low-risk patients, conservative surgery with adjuvant radiotherapy was associated with a lower disease progression risk and survival benefit compared to surgery alone [15].

In the present study, 5.6% of recurrences occurred among patients who underwent mastectomy, 38.8% among those treated with conservative surgery followed by radiotherapy, and 55.5% among those treated with isolated surgery, demonstrating worse outcomes in this low-risk VNPI classified group.

A systematic review by Goodwin et al. evaluated 3925 women with DCIS and observed ipsilateral and contralateral recurrence rates of 1–2%, lower than in our population, but similarly demonstrated worse outcomes for those treated with isolated conservative surgery. Patients who underwent mastectomy had recurrence rates of 0.5–1% per year in the contralateral breast, while those with conservative surgery had 5-year recurrence rates of 16%, reduced to 8% with radiotherapy addition [21].

The present data indicate that the VNPI was not an adequate stratification tool for our population, potentially underestimating risk for many patients. If classified by NCCN criteria, most would have been classified as high risk and managed with adjuvant radiotherapy or mastectomy. Among recurrences, 5.6% patients were under classified by VNPI and were not treated according to NCCN recommendations.

Although the NCCN guidelines’ emphasis on radiotherapy may reduce recurrence rates, it is important to consider potential long-term treatment-related morbidity, including radiation-induced fibrosis, lymphedema, and secondary malignancies. Future studies should evaluate whether the benefits of reducing recurrence outweigh these risks, particularly in low-risk patients.

In accordance, a retrospective study by Kunkiel and Niwinska evaluated VNPI’s prognostic value in 525 DCIS patients initially treated with lesion excision in a public hospital in Poland. Based on histopathological features of the specimen and VNPI risk, patients were triaged for observation, adjuvant radiotherapy, or mastectomy. After evaluating 5-, 10-, and 15-year recurrence-free survival rates, it was observed that individual risk groups did not differ significantly and VNPI was not an optimal tool for DCIS patients [11].

Throughout the study period (1996–2022), significant advancements in breast cancer management, particularly in ductal carcinoma in situ (DCIS) treatment, have been implemented. These include refinements in surgical techniques, radiation protocols, and systemic therapies, as well as improved imaging modalities and risk stratification tools. Given these changes, maintaining consistency in follow-up and outcome assessment presented a methodological challenge.

To address this, we stratified our analysis based on treatment era, ensuring that variations in clinical practice were accounted for. This stratification allowed us to compare recurrence rates within distinct periods, reducing bias introduced by evolving treatment standards. Additionally, we considered the impact of changes in diagnostic and therapeutic approaches on recurrence rates, acknowledging that newer strategies may lead to different clinical outcomes.

Despite these precautions, we recognize that our findings are influenced by institutional treatment guidelines and resource availability over time. Therefore, the conclusions drawn from this study should be interpreted in the context of our specific patient population and may not be directly generalizable to other healthcare settings.

Our study has some limitations inherent to observational designs, including potential inaccuracies in registries and inference limitations. Additionally, missing data due to loss to follow-up may bias results, particularly if related to the outcome.

Future perspectives in the treatment of DCIS increasingly focus on the role of genomic assays to refine patient stratification and personalize therapy. Tools such as the Oncotype DX DCIS Score [22] and the DCISionRT Decision Score [23] offer promising avenues to identify patients at higher or lower risk of local recurrence after breast-conserving surgery, thus guiding decisions on adjuvant radiotherapy and endocrine therapy. While these assays have shown potential in improving risk stratification beyond traditional clinicopathological factors, cost and accessibility remain significant barriers to widespread adoption, particularly in resource-limited settings. Furthermore, the predictive accuracy of these assays requires ongoing external validation in diverse patient populations to enhance clinical utility. Notably, prospective trials like the ELISA study are incorporating the Oncotype DX DCIS Score to define low-risk populations for whom radiotherapy may be safely omitted, marking a critical step forward in de-escalation strategies [24].

## 5. Conclusions

Our study provides an important comparative analysis of the VNPI and NCCN guidelines for the treatment of DCIS in a Brazilian cohort. The findings suggest that NCCN’s treatment recommendations may be associated with lower local recurrence rates when compared to VNPI-based decisions. However, our findings also highlight potential over-treatment concerns, given NCCN’s greater emphasis on radiotherapy. Kaplan–Meier survival analysis further supports these findings, showing no statistically significant difference in recurrence-free survival between invasive and in situ recurrences. Future prospective studies should further explore optimal risk stratification and treatment de-escalation strategies.

However, we recognize the limitations inherent to our retrospective study, particularly regarding the extended follow-up period and potential variations in clinical practices over time.

We acknowledge that our results are based on a single-institution database and may not be directly generalizable to other healthcare settings. Additionally, changes in breast cancer management from 1996 to 2022 necessitate a cautious interpretation of our findings. While our data provide valuable insights into the applicability of these guidelines, future prospective studies are needed to validate these results in broader, more diverse populations.

## Figures and Tables

**Table 1:** The Van Nuys Prognostic Index scoring system.

Feature	Score 1	Score 2	Score 3
**Size (mm)**	≤15	16–40	>40
**Margins (mm)**	≥10	1–9	<1
**Grade** **Necrosis**	Low or intermediate Without necrosis	Intermediate With necrosis	High grade With/without necrosis
**Age (years)**	>60	40–60	<40

Source: GORRINGE et al. [4].

**Table 2:** Descriptive statistics of quantitative variables in the study population.

Variable	Mean	Median	Standard Deviation	CV	Min	Max	n	95% CI
**Age (years)**	60,1	60	11,0	18%	32	91	116	58.1–62.10
**Follow-up (months)**	60,3	42	47,4	79%	6	203	116	51.67–68.93
**Time to Recurrence (months)**	20,9	14	17,6	84%	2	73	18	12.77–29.03

Legend: The confidence interval (CI) values refer to the respective variables of age, follow-up time, and time to recurrence. CV: Coefficient of Variation; Min: minimum/; Max: Maximum; CI: confidence interval.

**Table 3:** Clinical and pathological characteristics.

Variable		n	%	*p*-Value
**Menopause**	No	26	22.4%	<0.001
	Yes	90	77.6%	
**Family History**	No	100	86.2%	<0.001
	Yes	16	13.8%	
**Diagnosis**	Clinical Screening	28 88	24.1% 75.9%	<0.001
**Nuclear Grade**	Low	31	26.7%	0.050
	Intermediate	40	34.5%	
	High	45	38.8%	
**Comedonecrosis**	No	46	39.7%	0.002
	Yes	70	60.3%	
**Size (cm)**	<1.5 cm	64	39.7%	0.006
	1.6–4 cm	43	25.9%	
	>4 cm	9	6.9%	
**Margins (mm)**	<1 mm	36	29.2%	0.269
	1–9 mm	36	29.2%	
	>10 mm	44	41.6%	
**Hormonal Receptor**	Unknown	64	55.2%	0.008
	Positive	44	37.9%	
	Negative	8	6.9%	
**HER-2 Expression**	Unknown	64	55.2%	<0.001
	Positive	43	38.7%	
	Negative	8	6.9%	
**Treatment**	BCS e RT	50	43.1%	<0.001
	BCS	45	38.8%	
	Mastectomy	21	18.1%	
**Sentinel Lymph Node Biopsy**	Mastectomy	10	47.6%	<0.001
	BCS	5	5.2%	
**Endocrine Therapy**	No	104	89.7%	<0.001
	Yes	12	10.3%	
**Recurrence**	No	98	84.5%	<0.001
	Yes	18	15.5%	
**Recurrence-DCIS**	No	110	94.8%	<0.001
	Yes	6	5.2%	
**Recurrence-IBC**	No	104	89.7%	<0.001
	Yes	12	10.3%	

Original database//BCS, breast-conserving surgery; RT, radiotherapy; DCIS, ductal carcinoma in situ; IBC, invasive breast cancer.

**Table 4:** Distribution according to VNPI classification, treatment, and NCCN classification.

Classification	Treatment	n	%	*p*-Value
VNPI Low		45	38.8%	<0.001
VNPI Intermediate		62	53.4%	0.294
VNPI High		9	7.8%	<0.001
	BCS e RT	50	43.1%	<0.001
	BCS	45	38.8%	
	Mastectomy	21	18.1%	
NCCN High Risk		101	87.1%	<0.001
NCCN Low Risk		15	12.9%	<0.001

Original database//BCS, breast-conserving surgery; RT, radiotherapy.

**Table 5:** Recurrence according to NCCN- and VNPI-based treatments.

Guideline-Based Treatment		Recurrence		No Recurrence		Total		*p*-Value
		n	%	n	%	n	%	
NCCN	Yes	17	94.4%	84	85.7%	101	87.1%	0.310
	No	1	5.6%	14	14.3%	15	12.9%	
VNPI	BCS	10	55.6%	35	35.7%	45	38.8%	0.175
	Mastectomy	1	5.6%	20	20.4%	21	18.1%	
	BCS and RT	7	38.9%	43	43.9%	50	43.1%	

Original database//BCS-RT, breast-conserving surgery with radiotherapy; MT, mastectomy; BCS, breast-conserving surgery.

**Table 6:** Correlation of recurrence and VNPI and NCC subgroups.

Guideline Subgroups		Recurrence		No Recurrence		RR	95% CI	*p*-Value
		n	%	n	%			
NCCN	High	17	94.4%	84	85.7%	2.09	0.81–5.37	0.156
	Low	1	5.6%	14	14.3%	0.83	0.10–6.56	0.999
VNPI	Low	10	55.6%	35	35.7%	2.76	1.01–7.51	0.049
	Intermediate	5	27.7%	57	58.1%	0.36	0.13–0.99	0.049
	High	3	16.7%	6	6.2%	4.13	1.19–14.41	0.058

Original database//RR, relative risk; CI, confidence interval. The reference group for the relative risk (RR) analysis in the VNPI classification is the intermediate VNPI group. The RR values compare the risk of recurrence in the low VNPI and high VNPI groups relative to the intermediate VNPI group.

**Table 7:** Multivariate logistic regression analysis of recurrence predictors in DCIS patients.

Variable	Odds Ratio (OR)	95% CI Lower	95% CI Upper	*p*-Value
**Age (years)**	1.06	1.0	1.12	0.044
**Nuclear Grade**	1.16	0.55	2.44	0.701
**Tumor Size (cm)**	0.78	0.44	1.39	0.401
**Margin Status**	1.55	0.5	4.77	0.443
**Treatment**	0.62	0.18	2.13	0.447

Legend: Multivariate logistic regression model evaluating independent predictors of recurrence in patients with ductal carcinoma *in situ* (DCIS). The table presents odds ratios (OR) with 95% confidence intervals (CI) and *p*-values for each variable included in the model. Statistically significant values (*p* < 0.05) are highlighted in bold.

## Data Availability

The data are fully available from the author upon request.
